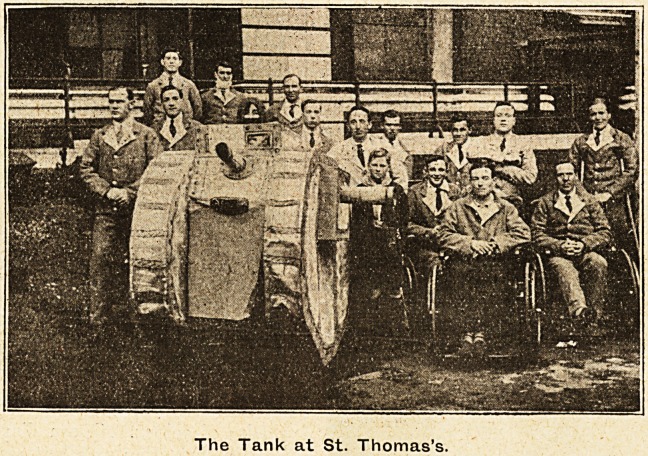# Bolo Buried and Forgotten Everywhere

**Published:** 1918-01-05

**Authors:** 


### OUR HOSPITALS' CHRISTMAS, 1917.

### BOLO BURIED AND FORGOTTEN EVERYWHERE.

### St. Bartholomew's Hospital.

By the Editor of its "Journal."

The Christmas festivities at "Bart.'s" were a great success and all concerned are to be congratulated on their efforts. The decorations compared very favourably with previous years, the soldiers' wards especially being greatly admired. A novelty in Charity Ward consisted of a model of a front-line trench complete with wire entanglements, a supporting line, model guns, trench mortars, communicating trenches, telephone and wireless stations. The Battalion, Brigade, and Divisional Headquarters were also represented. In this ward also several Tommies who had seen active service in East Africa had constructed an East African native kraal absolutely complete in every detail. In Harley and Kenton Wards several fracture cases had been put up with the Morris Sinclair apparatus, and the structures lent themselves admirably for decoration with evergreens, flags, Chinese lanterns, etc. Every ward in the hospital was cha[illegible]ingly decorated, and it would be invidious to make any distinctions.

Several concert parties toured the wards during the afternoon. Those taking part were: The Roland Ramblers, The Cheerohs, Miss Warren Fisher's Party, and last, but not least, the resident troupe, who described themselves as the "Bolos." The latter were particularly popular, and caused roars of laughter in their original costumes, consisting chiefly of operation gowns and bowler hats.

As in previous years a member of the junior staff, excellently got up as Father Christmas, distributed numerous presents to the various patients. Thanks to the foresight of the authorities all the patients were able to partake of the usual Christmas puddings, the ingredients for which were bought as far back as last October.

The usual resident staff dinner was given in the evening, after which there was a dance in the surgery. Subsequently the matron, warden, and steward were all visited in turn, the day closing with "Auld Lang Syne" in the hospital square. A word of thanks is due to the organisers of the War Emergency Concert Party under the direction of Mr. Isodore de Lara, which entertained the soldiers and patients so admirably on Boxing Day. A feature of the festivities this year was a children's party instituted by the Tommies, to which all the children in the hospital who could walk were invited. It is hardly necessary to say that this proved a tremendous success and constituted an excellent finish to a memorable Christmas.

The Tank at St. Thomas's.

### St. Thomas's Hospital.

At St. Thomas's Hospital Christmas has been marked as a period of quiet enjoyment and happiness. With nearly 500 military patients and over 400 civilians in their wards the staff resolved to make their patients feel happy. Throughout Christmas week the wards looked bright, and the faces of all showed happiness and contentment.

With a minimum of expense and a maximum of ingenuity, decorations of a simple kind were introduced into practically all the wards, the military wards and huts being made especially bright by the many little decorations contrived for the most part by convalescent soldiers. There were a few special entertainments, the first being a tea and variety show provided by the students of the massage department, who originally arranged a tea and one performace for the military patients who are under treatment there. This department has recently been considerably enlarged in view of the great increase of the work during the past year, and accommodation was found for over 300 men; but it was impossible to entertain all at one show, and therefore two "houses" in the most approved modern style were given on the one evening. A play had been carefully rehearsed, and went with a swing, the performers showing great skill in the parts they undertook. With instrumental music, songs, dances, and a most amusing Swedish drill which delighted the Tommies, a most enjoyable three hours was spent by all.

On Christmas Day the staff, sisters, and their friends had provided turkeys—there was but a slight alteration from the hospital routine—and the dinners wound up with the usual Christmas pudding. Teas and entertainments in the various wards, at which several well-known professional artistes kindly gave their services, concluded a very pleasant day; where, as one distinguished visitor remarked, he had seen more quiet happiness than anywhere else during this Christmas period.

On Boxing Day, and on the Thursday and Friday, the nurses, excellently trained, sang carols, and concerts were given in various wards. In Florence Ward, which is now devoted entirely to officers, a very excellent concert, to which many of the convalescent N.C.O.s and men from the other wards were invited, was given. This ward presented a particularly bright and cheery appearance from the efforts which had been made by the officers each to decorate his own "quarters." On the whole a most cheerful result was produced, many of the decorations showing real artistic merit.

**OUR HOSPITALS' CHRISTMAS, 1917.**—(*continued*).

In one of the huts a surprise was created by a very realistic Tank, made on the premises of very simple material, principally brown paper, and was a great success, so much so that it was promptly requisitioned to make a tour of the hospital, where it was greeted with loud applause on all hands. As one Tommy, a Newfoundlander, remarked, "Wouldn't that scare a German if he were a patient here?"

The week's festivities wound up with a great Christmas-tree provided for the casualty and out-patient children, of whom over 200 poor little mites were entertained at tea and provided with toys, their drawn faces sparkling with delight on the advent of Father Christmas and his attendant satellites, who quickly stripped the tree and distributed a toy to every child present.

---

### At "The London."

Queen Alexandra and Princess Victoria Present.

As compared with the Christmases of peace-time the Christmas of 1917 was very quiet indeed; it was, however, a very happy one. In peace-time there is a programme for every ward of entertainments which last from 2 to 7; the festivities have become so organised that perhaps they have lost a little of the spontaneity which is, after all, the charm of Christmas merry-making. This year every ward became its own family party. Sisters and nurses romped with their own children and did not depend upon the time-table visit of outside troupes. Perhaps the best feature of Christmas, and one new to this hospital, was the giving of a party to about 800 out-patient children in the big out-patient hall. Each child sat down to a hearty tea, permission for which was given by the Food Controller, and the evening was spent in cinemas, Punch-and-Judy shows, etc. Queen Alexandra and Princess Victoria delighted the children by their presence, and Her Majesty pulled crackers and joked with all she came across. Father Christmas, with ten or twelve attendants, dressed up as every nationality known to the schoolboy—and many that are not known—presented every child with a toy, a doll or a train, a book or a Teddy-bear, a box of soldiers or a Noah's Ark. These were not tawdry affairs made to give away, but were some of the beautiful toys sent by readers of the *Daily Mail*.

---

### Middlesex Hospital.

Christmas, that festival which everyone must still make bright for the children, and for those greater and no less light-hearted children, the Tommies, was, as usual, a gay and happy time at the Middlesex Hospital. The day was heralded by a choir composed of the nurses, who passed through every ward singing well-known carols, the charm and restfulness of their singing being a happy introduction for Christmas morning. Later the house surgeons and house physicians, arrayed in *recherché* female costumes, to which only the pen of a fashion-plate artist could do justice, and crowned with bewitchingly-dressed hair (which somehow suggested Clarkson), marched—one should rather say, minced—through each ward, extracting weird music from still more weird instruments, leaving behind them shrieks of amusement as each patient spotted his or her own "doctor" in that crowd of buxom wenches. At dinner-time, in accordance with a time-honoured custom, members of the honorary staff carved the turkeys each had given for his special ward, while their wives, the clerks, and dressers acted as waiters and helped to serve the dinner, with great success. Even the plum-pudding made its appearance, and was enthusiastically greeted as an old friend whose absence from the festive board had been regretfully anticipated. Every care was taken, however, to keep within the limits advised by the Food Controller.

In the afternoon toys from a beautiful Christmas-tree were distributed to the expectant children, who had long looked forward to this event. Their expectation had dated from the last air-raid, when they had been told that the "banging noise" all round them was Father Christmas collecting all his toys for Christmas. A splendid contribution to the tree had been sent by the Queen herself, who never forgets the small sufferers on that day. Useful gifts, too, for every patient were presented by friends of the hospital, who had generously raised a fund for the purpose. A pleasing feature of the afternoon, and one which probably gives the greatest joy of all to the patients, is that each may invite two friends to tea, and very few fail to make full use of this privilege. The wards this year had of necessity to be sparingly decorated, and very great ingenuity was shown by the Tommies, who evolved a wonderful scheme of decoration carried out and executed by themselves, which served as an excellent background for the attractive concert arranged for them from five to seven o'clock by the housemen and their friends. This closed the day, and, in the language of the immortal Pepys, "so to bed."

---

### St. Mary's Hospital.

There were two celebrations of Holy Communion in the morning, and carols were sung instead of the customary sermon. The chaplain presented each of the sisters with a little book of original poems. Every patient who was sufficiently well had turkey and plum-pudding for dinner (all food regulations were strictly adhered to). The turkeys were the gift of a kind friend. Smoking was allowed, each patient received a present and each child a toy.

The decorations were varied. Allcroft caught the eye of the visitor at once with its big red bell. Lewis Lloyd chose yellow and mistletoe, Lilian Holland yellow with life-like chrysanthemums, but Alexandra adhered to red. Each patient was allowed to ask a friend to tea at four o'clock. The doctors and students worked hard to give the patients a really good time. "Alice in Wonderland" was excellent, especially the Mad Hatter, Dormouse and March Hare. A piano was provided for every ward, and several ladies and gentlemen came in to sing and play, but local talent predominated. In short, everything went with a swing, and eight o'clock came all too soon.

---

### Metropolitan Hospital, Kingsland Road.

On Christmas Eve, as soon as the lights were out, nurses went round the wards carrying lamps and singing carols. All the wards were very prettily decorated; and there was keen rivalry between the several military wards. In many a regimental badge was placed in a position of prominence.

### OUR HOSPITALS' CHRISTMAS, 1917.—(*continued*).

On Christmas morning His Majesty the King's message to his wounded soldiers was read and received with rousing cheers. Each soldier patient received a box containing cigarettes, tobacco, a pipe, matches, and a 2s. book of stamps, all provided by the Haggerston and North-East London Hospital Aid Society. On the boxes were sprigs of holly prettily made by the girls employed at Mr. Corbett's artificial-flower factory. The Haggerston Hospital Society also gave twenty War Savings Certificates, which were drawn for amidst much excitement. The two house surgeons (dressed up as Father Christmas and clown) went round the wards during the morning and presented each patient—man, woman, or child—with a gift bought out of the matron's Christmas Fund, which also provided the Christmas dinner, turkey and plum-pudding. The toys and crackers presented to the children were collected by the Hon. Esmé Glyn, Lady Wolverton's youngest daughter, who is president of the Children's League; and there were besides many presents of tobacco, cigarettes, cakes, sweet, fruit, pipes, etc. The Mayor of Stoke Newington went round the wards at dinner-time, and spoke his kindly word to a large number of the patients. Each patient was allowed to have one visitor to tea and to the evening entertainment. Each visitor was requested to bring two lumps of sugar, which seemed to add to the general amusement, and produced the sugar.

In the military wards the patients were entertained with a Christmas-tree, games, and concerts; in the civilian wards the medical and nursing staff provided an excellent programme. The fairy tale "Cinderella," told by the matron, was enacted by her night nurses; and the home sister's troupe of day nurses gave a performance which included dancing, choruses, songs, and recitations. The sketches "A Sister to Assist 'Er" (by the doctors) and "'Tilda's New 'At" (by the matron, home sister, chaplain, and the sister of the out-patient department) were well received. Last, but not least, were the ever-popular "Mrs. Jarley's Waxworks," by the sisters. They have never failed to please, and this Christmas perhaps they were even better than usual. The matron's performances as "Mrs. Fishwick" in "'Tilda's New 'At," and as "Mrs. Jarley" in the waxworks, have caused many to wonder. But Miss Bennett's talents have undoubtedly been applied to their best use; and at the Metropolitan Hospital the intention is to "carry on" and keep alive, going well and strong.

### Whipps Cross Infirmary and War Hospital.

Christmas commenced by the singing of carols by the nursing staff on Christmas Eve. The nurses made a tour of the wards, commencing with the military annexe. Afterwards every patient had coffee and cake. This little attention is much appreciated and looked forward to by the patients on Christmas Day. There was early celebration in the chapel at six, seven, and eight, and morning service at 10.30. The services were well attended by the nurses and patients. Every child had a stocking, and every soldier a gift; much amusement was caused by a band of small boys, from the boys' ward, who were escorted by the night sister and paraded at an early hour round the military wards in improvised regimentals with varying musical instruments.

The wards all looked very bright and cheerful, with shades on the lights, holly, mistletoe, and flowers; the colour scheme being particularly pretty. One of the military wards had tiny gollywogs on the shades at each corner, these being made by the nurses during the morning. The matron and the medical superintendent, the chairman, and some members of the Committee went round the wards wishing everyone a happy Christmas. In the boys' ward the band performed at frequent intervals on a very cleverly constructed and gay bandstand, which stood in the centre of the ward. It was to this ward that the Queen and Princess Mary, after their visit, sent toys and a model Tank. These were much enjoyed by the children.

At twelve came the patients' dinner. The soldiers all had turkey (provided by contributions), plum-pudding, and mince-pies, the civilians roast beef and plum-pudding. The tables looked very effective, with table centres and flowers. There was plenty of music, as some of the wards had pianos, and all had gramaphones. Major Stewart and the matron received all members of the staff to tea from 4 to 6 P.M.

In the evening an impromptu concert was arranged by the soldiers and heard with great success. And so ended, as one of the patients said, "a perfect day."

On Boxing Day the nursing staff and maids had their dinner of roast beef and plum-pudding. In the afternoon a children's party was held in one of the wards. All the children who were well enough were there. Instead of a Christmas-tree a monster Tank, which had been made in the infirmary, stood in the centre of the ward, with plenty of room inside for the toys which Father Christmas (impersonated by one of the doctors) distributed after the children had had tea and pulled crackers. Later games were played. The "band," still in regimentals, were particularly boisterous. Many of the soldiers came over to see the fun, and helped in taking the little ones back to their respective wards. It was essentially to us all a soldiers' and children's Christmas, and the fact that they thoroughly enjoyed it was the one thing that mattered.

### Brompton Hospital for Consumption.
### The Children's New Year's Eve.

Through the kindness of the officers who are undergoing treatment at Lady Ridley's Hospital, the little patients, who form an important part of the work at Brompton Hospital, were enabled to spend a few hours of supreme enjoyment on New Year's Eve. All those attending the out-patient department were included in the invitation, and the joy in each home can be imagined as the mother received the postcard inviting her to bring her child to the feast and entertainment to be provided.

On arrival, many tables were seen laden with cake, bread and jam, and other simple fare, and full justice was done to tea. This was followed by the ever-popular Punch and Judy show, and after that there was a Christmas-tree laden with presents, each small guest receiving a gift at the hands of Father Christmas himself. At the conclusion of the entertainment, a spontaneous and hearty vote of thanks was accorded to the kind donors on the proposal of Lord Cheylesmore, the chairman of the hospital.

The further generosity of the officers at Lady Ridley's Hospital has enabled every patient in the hospital to have a New Year's Day dinner of roast turkey, which was also very greatly appreciated.

### OUR HOSPITALS' CHRISTMAS, 1917 —(*continued*).

### Bradford Royal Infirmary.

IN spite of the never completely dispelled feeling of strain and sadness due to the influence of the war, all at the Bradford Royal Infirmary did their utmost to make Christmas a time of joyousness to the patients and to the staff. The ward sisters spent a great deal of time and trouble in endeavouring to make the wards look as artistic as possible, and the decorations of evergreens, coloured paper, and fairy lamps served to do away with the atmosphere of austerity usually associated with a hospital ward. Early on Christmas morning Father Christmas, in the person of the R.S.O. (Dr. Rippiner), visited each individual patient and gave each one some useful gift. Later in the morning the Lord Mayor of the city, accompanied by a goodly company of friends of the hospital, made a tour of the wards. At mid-day the patients and staff were treated to a substantial Christmas dinner which had been provided by the Lord Mayor and other generous friends of the institution. In the afternoon a choir party from one of the churches of the city sang in all the wards, and a concert in the evening finished off a very happy day to all concerned. Throughout the week concerts were held in all the wards, and a very large array of artistes gave of their best for the benefit of the patients. On Boxing Day a fancy-dress dance was held in the out-patients' hall, and was a huge success. Many very pretty and original costumes were in evidence, and all those who take pleasure in the terpsichorean art enjoyed themselves to their hearts' content. On Thursday, Friday, and Saturday members of the nursing staff gave a variety entertainment which reflected great credit on all the performers as well as those who arranged and managed the production. The "show" was a great success, and was an eye-opener to all those who were not aware of the talent concealed under a nurse's uniform. A charge was made for admission, and a substantial sum was handed over to the "Comforts Fund" for the local war hospital. On the whole the Christmas festivities in 1917 were the best for many years, and will be looked back upon with pleasure by all those who from necessity or duty were obliged to spend Christmas at the Bradford Royal Infirmary.

### (Some) Leicester Hospitals.

A VISIT to the war hospitals of Leicester showed the Christmas spirit of gaiety and gladness to be as much in evidence as in former years—not perhaps so much in the decorations, for these were on a scale fitting to the anxious times, as in the great expectancy which was plainly observable on the faces of battle-scarred warriors back once again in Blighty. Their very eagerness to enjoy a full measure of Christmas sentiment was a great incentive to all associated with the hospitals to make the season a memorable one; for such it was in the Leicester institutions, where full programmes were arranged for the entertainment of the soldiers. In the larger hospitals the entertainments commenced on Saturday with concerts at the Base Hospital, the North Evington War Hospital, the Royal Infirmary, the Groby Road Hospital, and other local institutions. On this day, too, a commencement was made with ward decorations, the finishing touches to which were given on Monday. On Christmas Eve the united choirs of St. Peter's, St. James's, and St. John the Baptist Churches, accompanied by the military chaplains, made a tour of the wards of the Base Hospital and sang familiar Christmas carols in the wards. On Christmas Day Divine Service was held at 6 and 7 o'clock, and was followed at 11 A.M. by a parade service in the recreation-room. At 12.15 Christmas dinner was served, followed by dessert and cosaques provided by the lady visitors. Similar arrangements on Christmas Day were made at the North Evington War Hospital. At the Royal Infirmary Christmas fare was provided, followed by dessert, nuts, and crackers, and at 5 P.M. the nursing staff assembled for a perambulation of the wards for carol-singing. At the Groby Road Hospital dinner was succeeded by a concert arranged by Mr. W. H. Brain. The evening was passed at most of the institutions in whist drives and competitions which the soldiers are quite experts in organising.

These programmes were repeated in a fuller measure on Boxing Day—the soldiers themselves in some of the institutions arranging musical programmes and ward competitions. The annual Christmas entertainment at the Base Hospital was given on the evening of Boxing Day by well-known local entertainers. In addition to the usual supplies of tobacco and cigarettes the local War Hospitals Committee made liberal presentations of tobacco and supplies, which were much appreciated. These extra supplies were possible through a generous gift of £50 from the War Fund of the employees of Messrs. M. Wright and Sons, Ltd., of Quorn. In a subsequent issue we hope to give particulars of entertainments at Leicester institutions.

### Norfolk and Norwich Hospital.

ALTHOUGH no appeal was made to the public for funds to purchase extra comforts, and gifts were less numerous than usual, there is no doubt that all the patients had a good time this Christmas. A sharp frost and a snow-covered ground did not prevent a party of nurses rising at 4.30 in order that the time-honoured custom of commencing Christmas Day by the singing of carols around the wards at 5 A.M. should be maintained. The wards were tastefully decorated, a spirit of emulation amongst the patients of the various military wards adding to the usual scheme of decoration. Celebrations of Holy Communion in the chapel at 6 A.M. and 8.30 A.M. and a short service at 10.30 A.M. were well attended both by the staff and patients. At 12.15 the patients' dinners were served. Owing to the kindness of generous donors there were sufficient turkeys from which all in the hospital were able to enjoy goodly portions, and, as usual, the birds were carved by members of the honorary and resident medical staffs. The puddings which followed were of excellent quality and greatly enjoyed. Crackers were pulled, and the articles produced from them added to the fun and merriment which prevailed. Smokes for the soldiers were abundantly supplied by the patrons of the London Palladium and a member of the board of management.

The recreation hall was crowded at 3.30 P.M., when an admirable concert was given by members of the hospital staff and the military patients. Many enjoyable entertainments have been given in this hall during the two years of its existence, but seldom has enthusiasm run so high as on Christmas afternoon. A party of entertainers, well known in the City, had arranged to provide the concert, but had to cancel the engagement a few days before Christmas, so that a few members of the nursing staff are to be warmly congratulated on discovering such excellent talent in the hospital at such short notice. After tea, the patients proceeded to enjoy what was termed on the timetable for the day as a "social evening in the wards." The staff of each ward entered with zest into the general fun provided by the patients, and music and games made the evening pass almost too quickly. In the military wards the fun was uproarious, but never exceeded the bounds of reasonable discipline. At 9 P.M. the proceedings came to an end, all the patients retiring to bed, many of them tired, but all very happy. The military patients included a large number of Colonials, many of whom were spending their first Christmas in England. Great was their anxiety to secure souvenirs of the hospital festivities to send to their friends in the dominions overseas. The general opinion can be summed up in an expression used by a Colonial soldier patient, who, being commiserated upon the fact that he was spending his fourth Christmas away from his native land, replied that "the next best place to home in which to spend Christmas was at the Norfolk and Norwich Hospital."

**[We regret that pressure on our space compels us to hold over other accounts and several articles.]**

## Figures and Tables

**Figure f1:**